# Trickier than It Looks: Isomerization between Five- and Six-Coordinated Zinc in Heterometallic Li_2_Zn_2_ Molecule

**DOI:** 10.1021/acs.inorgchem.4c00634

**Published:** 2024-06-21

**Authors:** Yuxuan Zhang, Haixiang Han, Zheng Wei, Evgeny V. Dikarev

**Affiliations:** † Department of Chemistry, University at Albany, Albany, New York 12222, United States; ‡ School of Materials Science and Engineering, Tongji University, Shanghai 201804, China

## Abstract

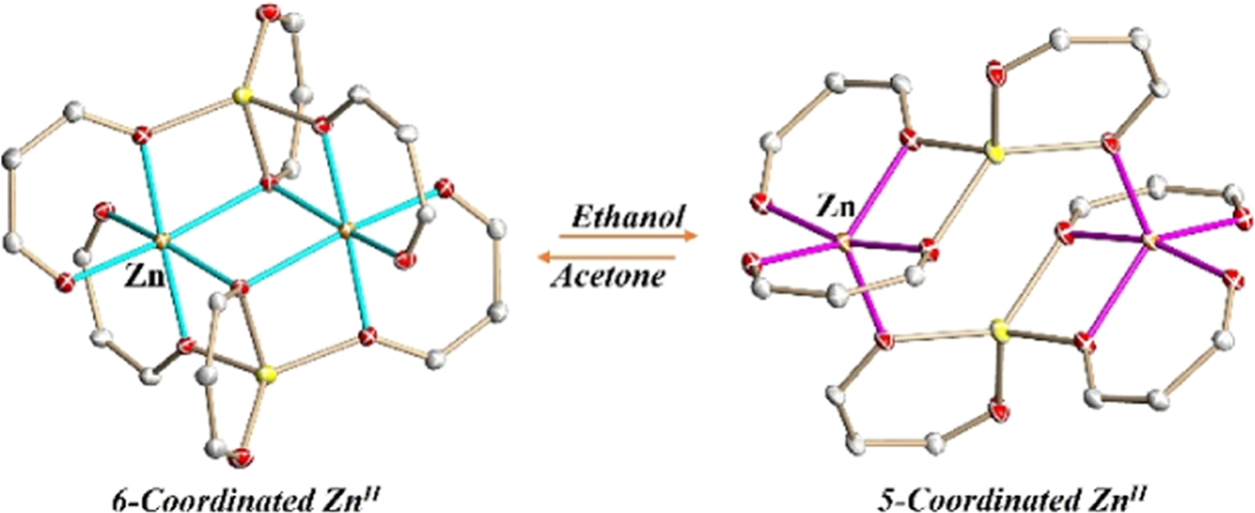

This report describes the synthesis and characterization of two hetero*bi*metallic Li–Zn coordination isomers [Li_2_Zn_2_(tbaoac)_6_] (tbaoac = *tert*-butyl acetoacetato) that have been isolated separately by the same stoichiometric reaction run in different organic solvents. The 6-coordinated zinc isomer (**6-Zn**) was synthesized in acetone with high yield, while the 5-coordinated one (**5-Zn**) was readily obtained from ethanol. The **5-Zn** isomer has a low solubility in organic solvents such as alkanes and haloalkanes, while its **6-Zn** counterpart exhibits a good solubility in almost all common solvents. Two isomeric molecules feature similar centrosymmetric tetranuclear cyclic assemblies, which are different in their arrangement of tbaoac ligands. While all ligands act as μ_2_-type in the structure of **5-Zn**, the two tbaoac groups chelating Li appear as μ_3_-type in **6-Zn**, thus providing an additional coordination for Zn ions. However, the real structural transformation between these isomers was shown to be more complex than simply making or breaking a couple of Zn–O bonds. X-ray single-crystal structure analysis, powder X-ray diffraction, multinuclear NMR, DART mass spectrometry, ICP-OES analysis, and TGA have been employed for the characterization of the isomers. The combination of powder X-ray diffraction and ^1^H NMR investigation revealed that **6-Zn** isomer can be quantitatively transformed to **5-Zn** in ethanol, while the reverse conversion instantly takes place in acetone.

## Abstract 2

Two coordination isomers of the heterometallic tetranuclear cyclic assembly [Li_2_Zn_2_(tbaoac)_6_] (tbaoac = *tert*-butyl acetoacetato) have been isolated. It has been found that 6-coordinated Zn can quantitatively isomerize to 5-coordinated Zn in ethanol, while the latter rearranges back in acetone.

## Introduction

Zinc is a vital element involved in a wide variety of structures ranging from simple coordination compounds to biosupramolecules and MOFs containing Zn(II).^1−9^ The d^10^ configuration is intertwined with a flexible coordination environment so that geometries of Zn(II) ion can vary from tetrahedral to trigonal bipyramidal and square pyramidal to octahedral.^5^

In biochemical studies, as a resident of more than 300 enzymes,^10^ zinc is known to be indispensable for the transmission and development of genetic messages by acting in numerous binding sites to orchestrate almost all aspects of metabolism, typically featuring coordination numbers of 4 or 5.^11,12^ Common bioinorganic processes such as alkaline phosphatase or carbonic anhydrase, to name a few, are known to be driven by zinc enzymes with flexible metal coordination revealed by proposed mechanisms.^13,14^

Zn-based MOFs employed in multiple chemical transformations and contributed to the studies of drug delivery systems and biosensors^15^ were found to be effective due to a great adaptability of Zn geometry for catalytic actions.^16−20^ To model these reactions, a number of zinc(II) coordination complexes with different coordination numbers have been synthesized and studied for mimicking and gaining a molecular-level understanding of the mechanisms behind.^21−26^ Such Zn(II) model systems from simple mononuclear complexes to more sophisticated designs featuring multiple metal centers functionally simulate the active sites of the enzymes and macromolecules.^27^

In a number of reports on zinc(II) complexes, coordination isomers with the same molecular formula but with different arrangements of ligands around the central metal ion^28^ have been shown to contribute to various mechanistic studies.^29,30^ That said, reports on interconversion between 4-, 5-, and 6-coordinated Zn(II) isomers are very rare among numerous zinc complexes. To the best of our knowledge, the transformation between two Zn(II) coordination isomers was mainly illustrated in the catalytic cycles with one of the isomers proposed as a transition state.^31−33^

Coordination numbers of zinc(II) ion in its complexes with primarily chelating ligands were also found flexible.^34,35^ Thus, 2D polymeric framework [Zn_2_(BDC)_2_L(H_2_O)_2_]_*n*_ (BDC = benzenedicarboxylate; L = *N*,*N*′-di(2-pyridyl)adipoamide) exists in the form of two isomeric structures with 4- and 5-coordinated Zn, imposed by the ligand isomerism.^36^ The [Zn(DBM)_2_] (DBM = dibenzoylmethanate) appears in two isomeric forms as either a 4-coordinated Zn monomer or a 5-coordinated dimer.^37^ Complex [Zn(bipy)(DBM)_2_] was shown to crystallize from different solvents as a dimeric, trimeric, or polymeric assembly while featuring 5- and/or 6-coordinated Zn ions.^38^

In this study, we describe the synthesis, properties, structures, and isomerization of a pair of zinc(II) coordination isomers of the heterometallic tetranuclear assembly [Li_2_Zn_2_(tbaoac)_6_] (tbaoac = *tert*-butyl acetoacetato). While the change in the bridging connectivity of two tbaoac ligands from μ_3_- to μ_2_-type simplistically explains the structural connection between two isomers, the real transformation between these molecules appears to be more complex than simply breaking a couple of Zn–O bonds. The isomers were found to have some distinctively different properties, and the isomerization process was confirmed in solutions of coordinating solvents.

## Results and Discussion

### Synthesis and Properties

**Figure 1:**
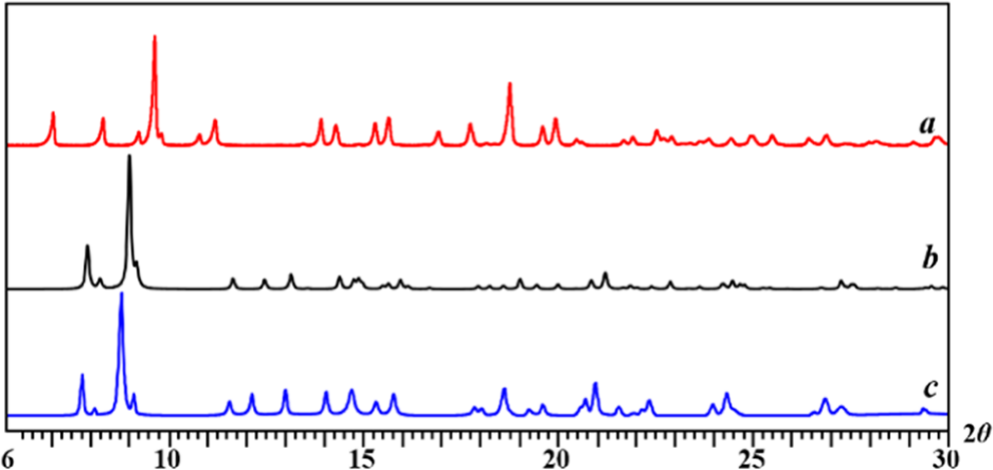
Powder X-ray diffraction patterns of (a) precipitate obtained in ethanol (**5-Zn**); (b) calculated powder pattern of [Li_2_Co_2_(tbaoac)_6_]; and (c) product obtained in acetone (**6-Zn**).

Heterometallic complexes [Li_2_M_2_^II^(tbaoac)_6_] (M^II^ = Mg, Fe, Co, Ni, and Co/Ni) have been reported as prospective single-source precursors for the Li/M = 1:1 oxide cathode materials.^39,40^ These tetranuclear assemblies featuring different divalent metal ions were found to be isomorphous and exhibit similar physical and chemical properties. However, this similarity came to an end upon the investigation of the Zn^II^ analogue. Following the same preparation procedure of adding stoichiometric amounts of Li(tbaoac) to anhydrous ZnCl_2_ in ethanol (eq 1) and stirring it for 12 h at room temperature surprisingly generated a large amount of white precipitate, which contradicted our previous observations of [Li_2_M_2_^II^(tbaoac)_6_] complexes having a high solubility in alcohols. The powder X-ray diffraction pattern of the white precipitate (Figure 1a) was also completely different from those of [Li_2_M_2_(tbaoac)_6_] compounds (Figure 1b). Conversely, reaction 1 performed in acetone or THF did not generate any precipitate, and the residue obtained upon removal of solvent and LiCl byproduct showed an X-ray powder diffraction pattern (Figure 1c) similar to known [Li_2_M_2_(tbaoac)_6_] complexes.
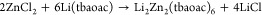
1

While the reaction products obtained in ethanol and acetone displayed completely different powder X-ray diffraction patterns, the ICP-OES analysis (see the Supporting Information, page S4) verified the Li/Zn ratio of 1:1 for both. Single-crystal X-ray diffraction investigation (vide infra) confirmed that the products are isomers differed by coordination of the Zn ions that we designate as **5-Zn** (5-coordinated Zn obtained in ethanol) and **6-Zn** (6-coordinated Zn isolated from acetone, analogous to all previously reported [Li_2_M_2_(tbaoac)_6_]).

Both **5-Zn** and **6-Zn** are stable in the presence of oxygen and retain their crystallinity in moist air for a few days. The **5-Zn** isomer has poor solubility in methanol, ethanol, and weakly coordinating solvents such as chloroform or dichloromethane, while practically insoluble in noncoordinating solvents such as hexanes, pentanes, and toluene at room temperature. However, the **5-Zn** is soluble in ketones (acetone or pinacolone) as well as in strongly coordinating solvents such as H_2_O and DMSO. On the other hand, the **6-Zn** isomer is soluble in almost all common organic solvents at room temperature, saving poor solubility in diethyl ether. Both isomers are not volatile under static vacuum conditions (sealed evacuated ampules) and start to show traces of decomposition when the temperature is raised to 120 and 150 °C, respectively. The phase purity of bulk products was checked by the powder X-ray diffraction, and the Le Bail fit was performed to confirm that the experimental powder patterns of **5-Zn** and **6-Zn** products correspond to the theoretical spectra calculated from the single-crystal X-ray data (Figures S1 and S2 and Tables S1 and S2).

### Solid-State Structures of **6-Zn** and **5-Zn** Isomers

**Figure 2:**
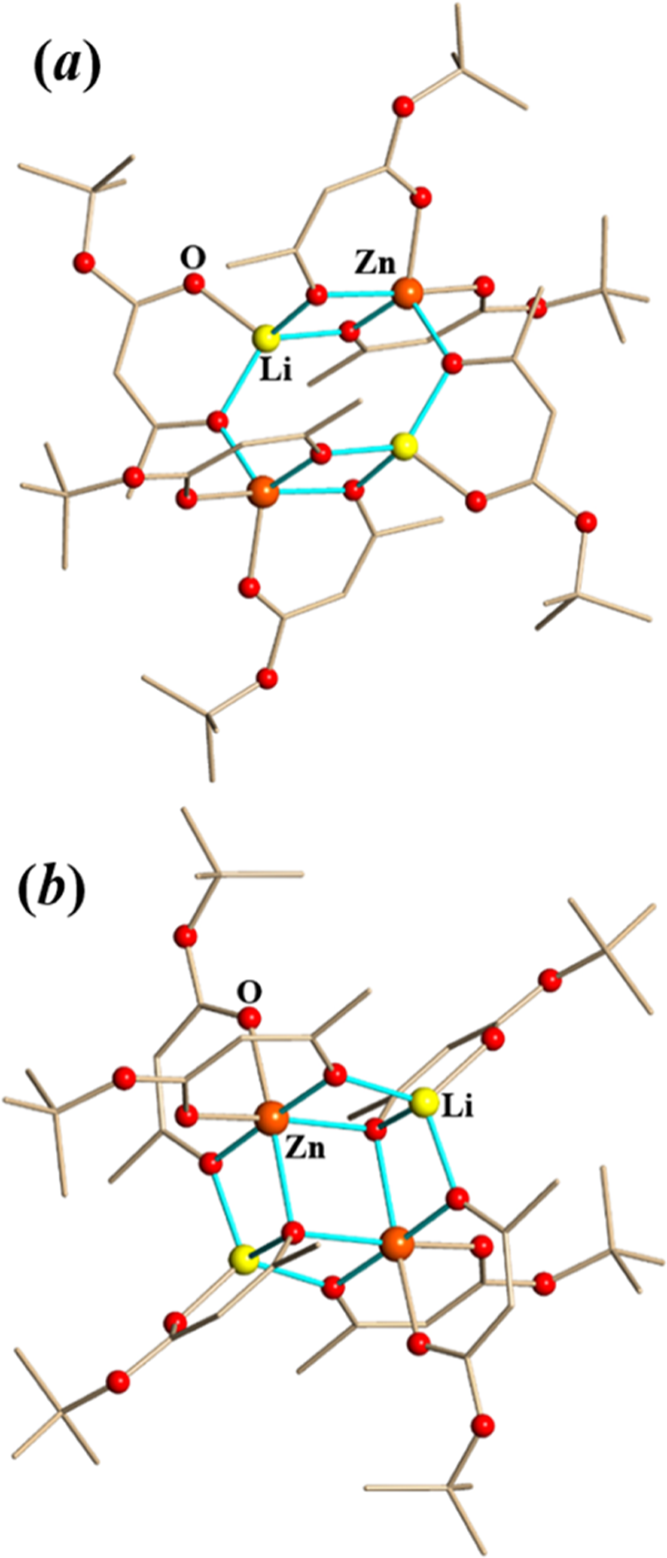
Solid-state structures of (a) **5-Zn** and (b) **6-Zn** isomers. The Li–O and Zn–O bonds to the tbaoac ligand oxygens involved in bridging interactions are shown in blue. Hydrogen atoms are omitted for clarity. The full views of the structures drawn with thermal ellipsoids can be found in the Supporting Information, Figures S3 and S4.

Two products from reaction 1 were crystallized from dichloromethane (**5-Zn**) and acetone (**6-Zn**), and the single-crystal X-ray data refinements revealed a pair of coordination isomers with the formula [Li_2_Zn_2_(tbaoac)_6_] (Figure 2). The **6-Zn** complex (Figure 2a) with two 6-coordinated Zn centers is an analogue to the previously reported [Li_2_M_2_(tbaoac)_6_] (M = Fe, Co, Ni, and Mg),^39^ while **5-Zn** (Figure 2b) featuring two 5-coordinated Zn ions has never been seen before with other metals. Both isomers are built of tetranuclear cyclic assemblies with only half of the molecules being crystallographically independent. In both molecules, one tbaoac ligand chelates Li ions, and two ligands chelate Zn ions. In each tbaoac ligand, only the oxygen atom beneath the methyl substituent is participating in bridging interactions, while the other one, under the bulky *tert*-butyl group, remains simply chelating. In both structures, the Zn centers are chiral (Figure S5), though the tetranuclear molecules are *meso* due to the inversion center in the middle of the assemblies.

**Figure 3:**
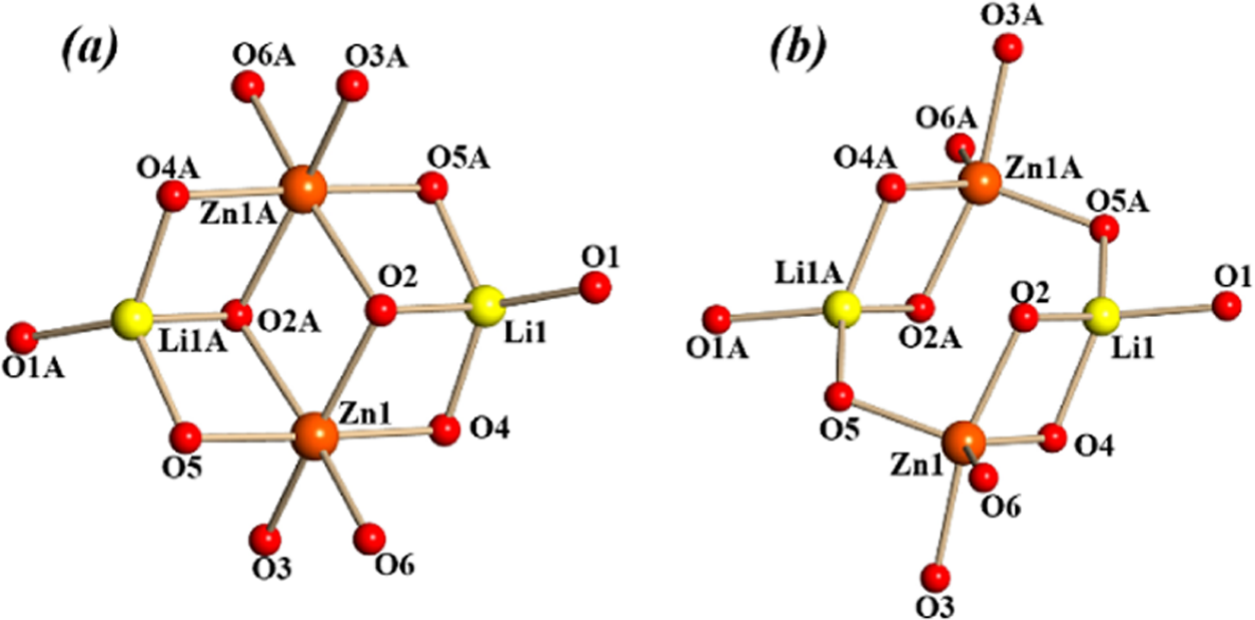
Simplified relationship between the structures of (a) **6-Zn** and (b) **5-Zn**. Only the lithium, zinc, and oxygen atoms are shown.

**Figure 4:**
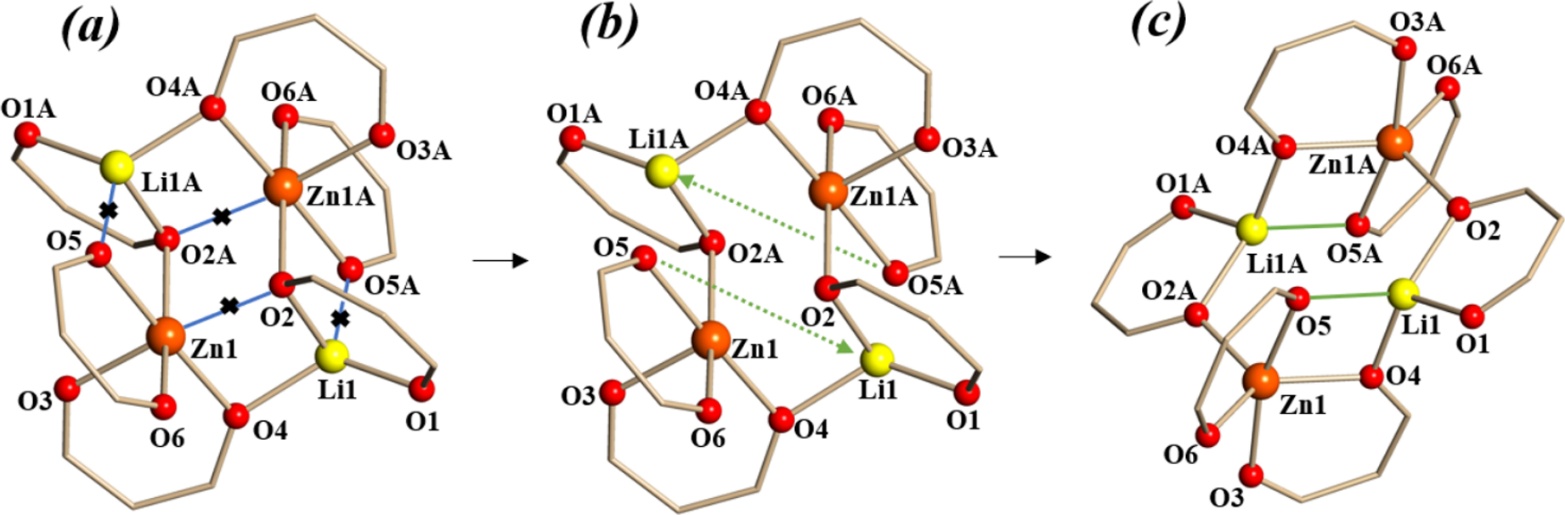
Schematic illustration of the structural transformation between **6-Zn** and **5-Zn** isomers.

The obvious difference between the two isomeric structures comes from the two tbaoac ligands chelating Li centers. In the **6-Zn**, these two groups act as μ_3_-type by chelating to Li and bridging to two Zn ions. In the **5-Zn** molecule, these ligands are both μ_2_-type bridging to only one Zn ion instead, eventually making Zn centers as 5-coordinated. At first sight, it is tempting to rationalize the relation between two isomeric tetranuclear assemblies by simply breaking two bonds Zn1(Zn1A)–O2A(O2) as shown in Figure 3, accompanied by the structure rotation around the Zn1–Zn1A axis. However, detailed analysis revealed that the transformation between **6-Zn** and **5-Zn** isomers is very complex. It involves (at least) breaking four M–O bonds and making two new M–O connections. The schematic illustration of this process is shown in Figure 4. First, two Li1(Li1A)–O5A(O5) bridging bonds and two Zn1(Zn1A)–O2(O2A) bridging bonds should be cleaved (Figure 4a,b). This release of stress should be followed by significant stretching and rotation of the structure to allow for new Li1(Li1A)–O5(O5A) bridging connections to be made (Figure 4b,c).

Table 1 shows the corresponding M–O distances in two isomers, specifically emphasizing the breaking of Zn1–O2 and Li1–O5A and making new bridging Li1–O5 bonds. As expected, the change of coordination number made the average Zn–O bond distance in **5-Zn** shorter than that in **6-Zn**, while the corresponding trend in the Li–O distances is the opposite. In both structures, two Li and two Zn atoms are in the same plane sitting at the vertexes of a rhombus. In that sense, the isomerization from **6-Zn** to **5-Zn** can be regarded as stretching the former structure along the Zn1···Zn1A axis (from 3.299(3) to 4.960(3) Å) as well as compressing it along the Li1···Li1A axis (from 4.776(3) to 3.842(2) Å), thus dramatically changing the shape of the rhombus characterized by the angles Li1···Zn1···Li1A and Zn1···Li1···Zn1A (Table 1).

**Table 1:** Selected Distances (Å) and Angles (deg) in the Structures of **6-Zn** and **5-Zn** Isomers

distances (Å)		**6-Zn**	**5-Zn**
Zn–O^a^	Zn1–O2	2.1487(12)	4.609(3)
	Zn1–O2A	2.1805(12)	1.9956(7)
Zn–O^b^	Zn1–O3	2.0400(12)	2.0728(7)
	Zn1–O6	2.0466(13)	2.0177(6)
Zn–O^c^	Zn1–O4	2.0254(12)	1.9988(7)
	Zn1–O5	2.0214(12)	2.0472(7)
Zn–O_av._		2.0771(12)	2.0264(7)
Li–O^a^	Li1–O5A	1.883(3)	3.497(3)
	Li1–O5	4.567(3)	1.9641(18)
	Li1–O4	1.872(3)	1.9216(19)
Li–O^b^	Li1–O1	1.842(3)	1.8975(19)
Li–O^c^	Li1–O2	1.938(3)	1.9293(18)
Li1–O_av._		1.884(3)	1.9281(18)
Zn1···Zn1A		3.299(3)	4.960(3)
Li1···Li1A		4.776(3)	3.842(2)

angles (deg)		**6-Zn**	**5-Zn**
Li1···Zn1···Li1A		75.48(7)	110.73(5)
Zn1···Li1···Zn1A		104.52(7)	69.27(5)

a Bridging oxygen.

b Chelating oxygen.

c Chelating–bridging oxygen.

### Characterization of **5-Zn** and **6-Zn** Isomers

**Figure 5:**
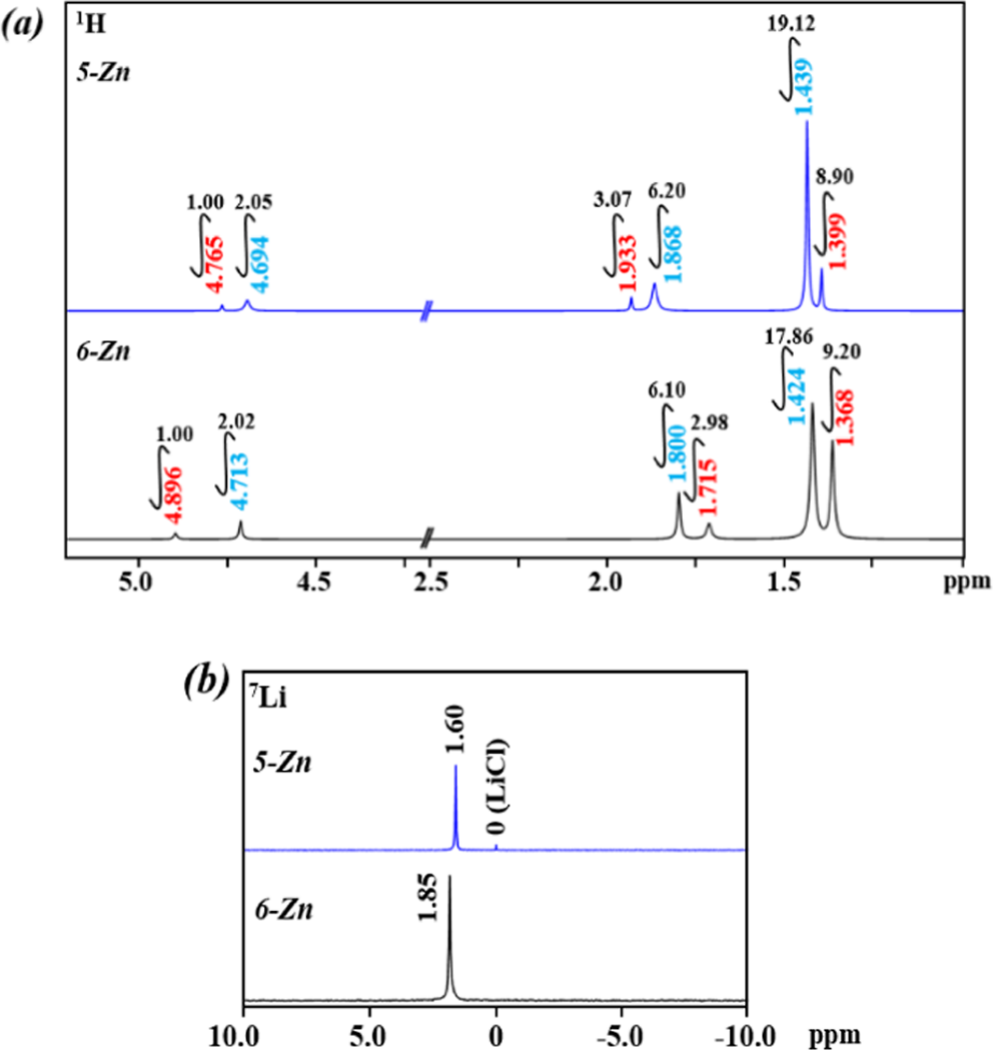
(a) ^1^H and (b) ^7^Li NMR spectra of **5-Zn** and **6-Zn** isomers in CDCl_3_ recorded at room temperature.

Examining solutions of **5-Zn** and **6-Zn** in noncoordinating solvents such as hexanes, dichloromethane, and chloroform (even with low solubility of the former isomer) revealed a number of important features. First of all, there is no decomposition, transformation, or isomerization taking place in these solvents based on continuous monitoring of the NMR spectra. The crystals of both **5-Zn** and **6-Zn** can be readily grown from the corresponding solutions. No ^1^H or ^7^Li NMR signals of Li(tbaoac)^40^ indicating the breakdown of heterometallic assemblies were detected. Importantly, the ^1^H NMR spectra of **5-Zn** and **6-Zn** (Figure 5a) correspond to the solid-state structures of the isomers described above. Both spectra show two sets of proton signals in a 1:2 ratio corresponding to two different coordination environments of tbaoac ligands primarily chelating Li and Zn ions, respectively. Each set of signals has three singlet peaks, matching for ligand −C*H*, −C*H*_3_, and −OC(C*H*_3_)_3_ protons in ca. 1:3:9 ratio. The ^7^Li NMR spectra (Figure 5b) of the isomers both display one signal with different chemical shifts.

**Figure 6:**
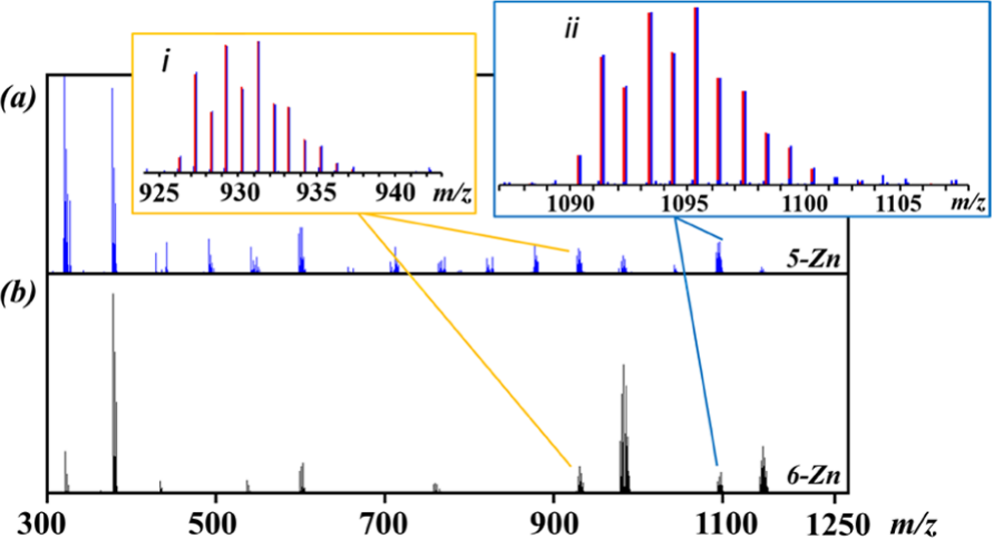
Positive ion DART mass spectrum of (a) **5-Zn** and (b) **6-Zn**. The isotope distribution patterns of the (i) [M-tbaoac]^+^ and (ii) [M+Li]^+^ ions are shown in the inset (M = [Li_2_Zn_2_(tbaoac)_6_]). Red and blue bars are calculated and experimental data, respectively.

Direct Analysis in Real Time (DART) mass spectrometry investigation unambiguously revealed the presence of the heterometallic species in the gas phase, confirming the retention of heterometallic molecules of **5-Zn** and **6-Zn** upon evaporation (Figure 6). For both isomers, [M – L]^+^ (M = [Li_2_Zn_2_(tbaoac)_6_]; L = tbaoac) meas/calcd = 930.605/930.608 and 930.612/930.608 and [M + Li]^+^ meas/calcd = 1094.743/1094.735 and 1094.720/1094.735 ions can be clearly identified in the positive mode spectra, showing excellent agreement with their respective calculated isotope distribution patterns. The **5-Zn** appears significantly more fragmented than **6-Zn** at the same experimental conditions (Figure 6), which can be used for recognition of the isomers. A full set of fragment ions is shown in Tables S6 and S7.

**Figure 7:**
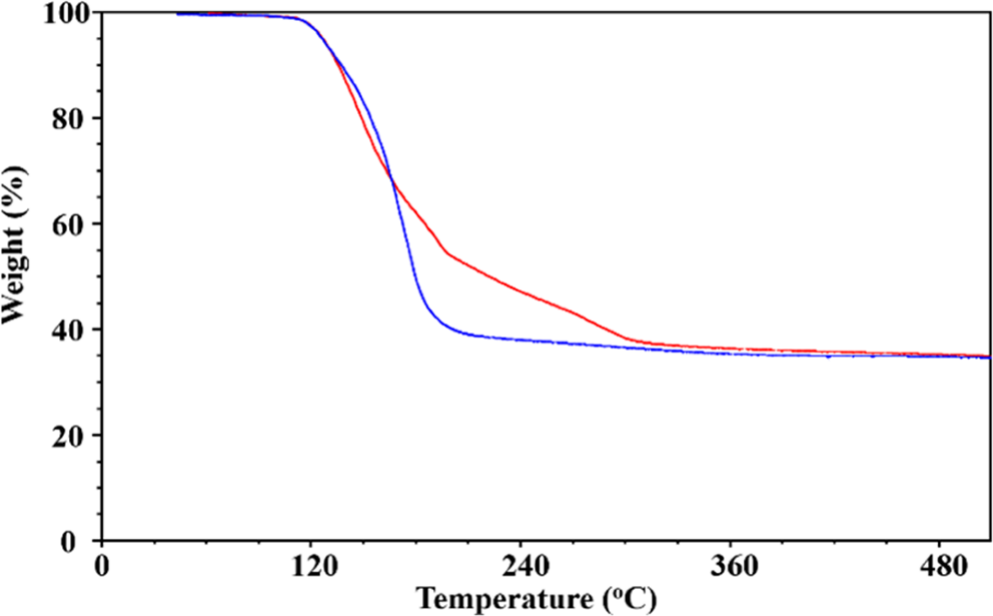
TGA plots of **5-Zn** (blue) and **6-Zn** (red) isomers.

TGA analysis (Figure 7) revealed that **5-Zn** and **6-Zn** isomers start to decompose at around 135 °C under a 25 mL/min N_2_ flow and share a similar decomposition pattern. The **5-Zn** is losing weight faster and at lower temperatures than **6-Zn**, in accordance with mass spectrometry observations that the former structure is easier to be destroyed. X-ray powder diffraction analysis of the residues for **5-Zn** and **6-Zn** samples after TGA measurements confirmed the presence of LiO, Li_2_CO_3_, and ZnO. No known mixed-metal Li–Zn oxide phases, such as Li_2_ZnO_2_,^41^ were found in the decomposition traces.

### Isomerization of **5-Zn** and **6-Zn** Molecules

Studies of the possible interconversion between two isomers have been carried out in both solid-state and solution environments. Since both isomeric forms are not volatile either in a dynamic or in a static vacuum, no isomerization can be accounted for in the gas phase. Similarly, there is no transformation between **5-Zn** and **6-Zn** happening in the solid state (crystal-to-crystal) as that was monitored by the X-ray powder diffraction technique upon heating both isomers at the temperatures close to the decomposition points for prolonged time under anaerobic conditions. As it was mentioned before, there is also no isomerization observed in solutions of noncoordinating solvents, such as hexanes, ethers, and chlorohydrocarbons, confirmed by continuous measurements of proton NMR spectra, as well as by analysis of the crystallization products.

**Figure 8:**
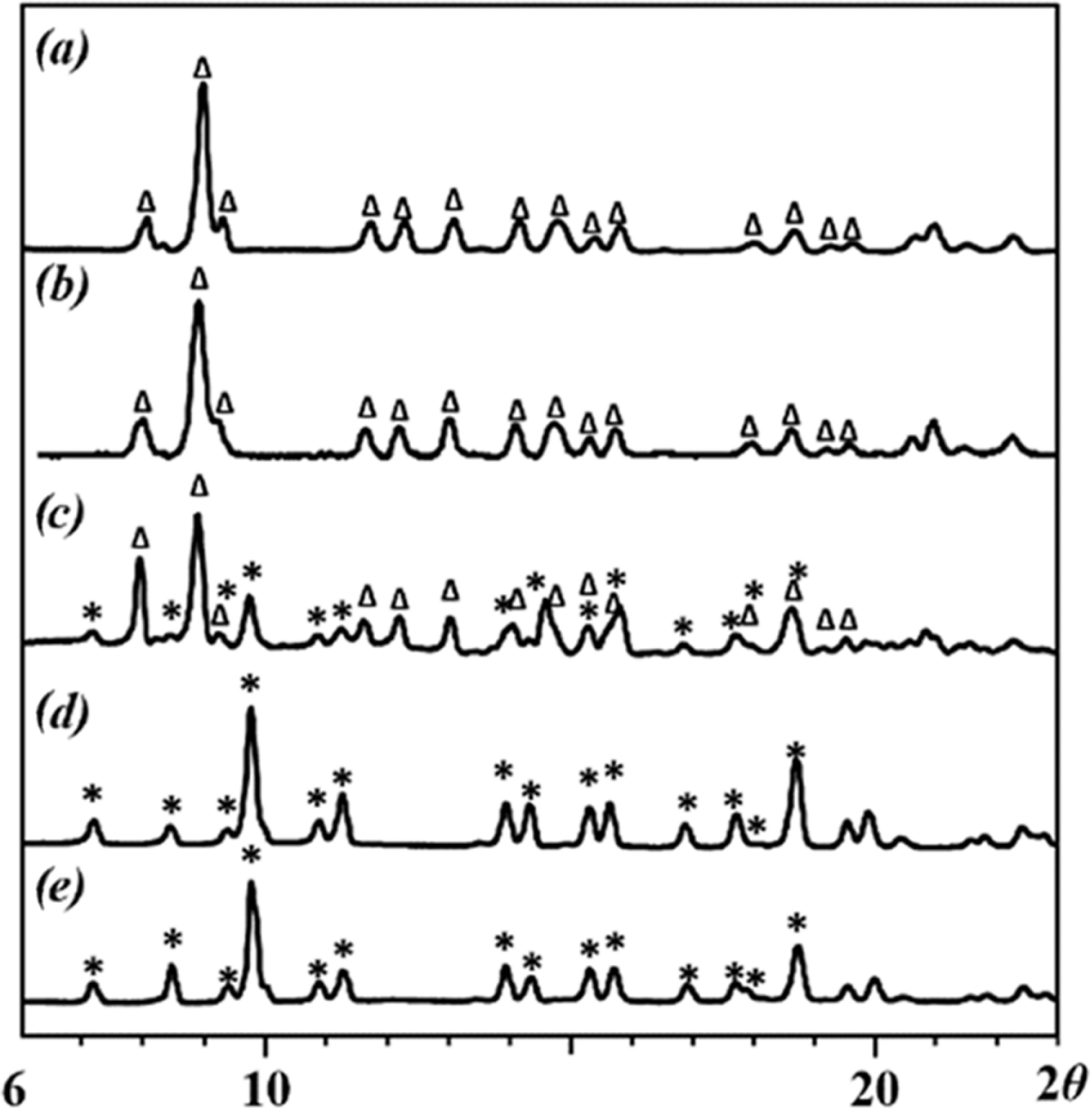
X-ray powder diffraction patterns of residues obtained upon evaporation of solvent after dissolution of **6-Zn** isomer at room temperature in ethanol at different times: (a) **6-Zn** upon dissolution; (b) after 1 h; (c) after 6 h; (d) after 2 days; (e) **5-Zn** upon dissolution. The Δ and * labels designate **6-Zn** and **5-Zn** theoretical peak positions in powder X-ray diffraction patterns between 2θ = 6 and 20°, respectively.

**Figure 9:**
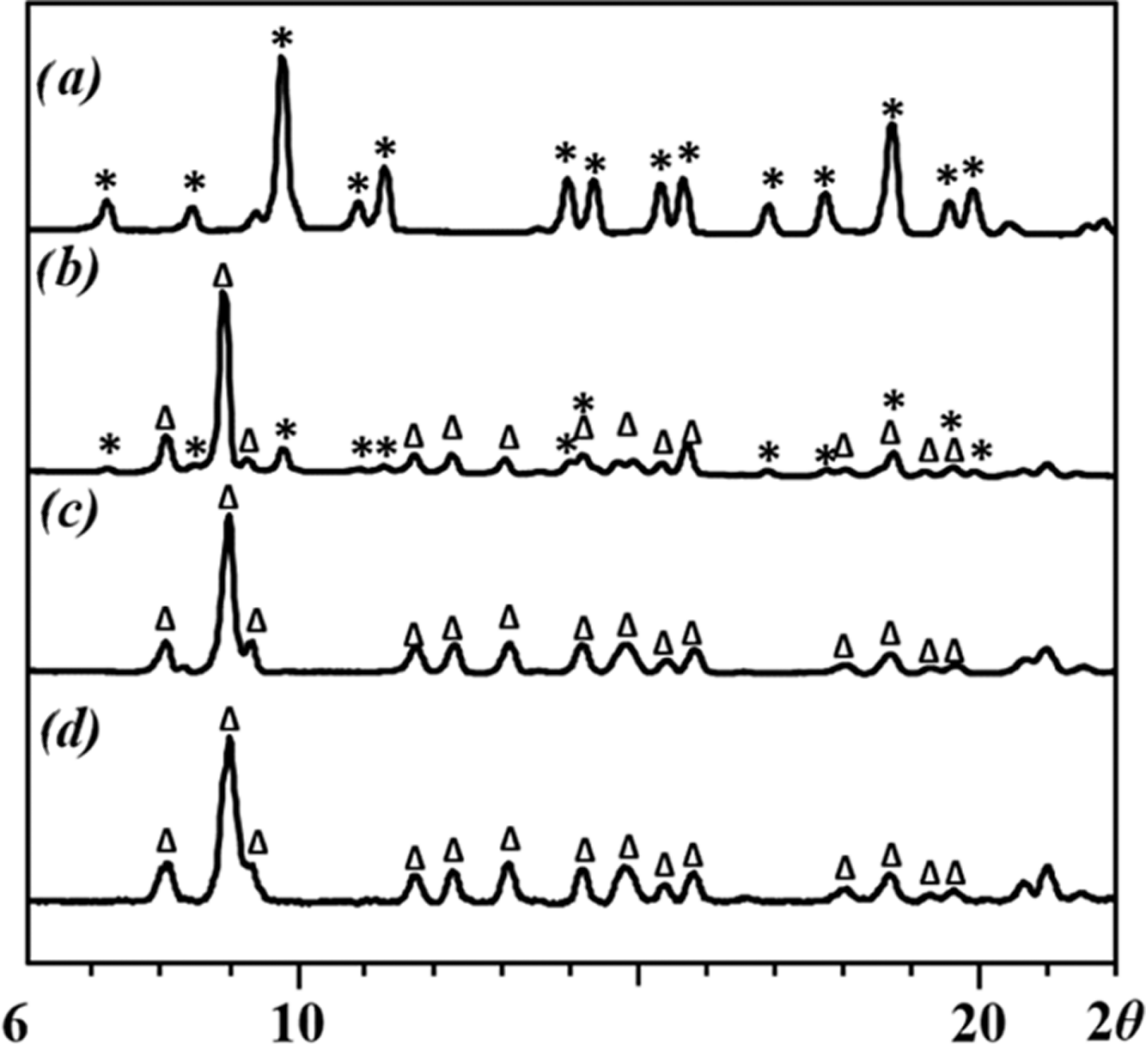
X-ray powder diffraction patterns of residues obtained upon evaporation of solvent after dissolution of **5-Zn** isomer at room temperature in acetone at different times: (a) **5-Zn** upon dissolution; (b) after 30 min; (c) after 3 h; (d) **6-Zn** upon dissolution. The Δ and * labels designate **6-Zn** and **5-Zn** theoretical peak positions in X-ray diffraction patterns between 2θ = 6 and 20°, respectively.

The transformation between the **5-Zn** and **6-Zn** isomers clearly takes place only in solutions of coordinating solvents. Those do not include “strongly” coordinating solvents (H_2_O, DMSO) since the latter were found to irreversibly cleave the heterometallic assemblies (Figure S6). The powder X-ray analysis of the solid residues obtained by evaporating the solvents after different times unambiguously confirmed the complete transformation of **6-Zn** into **5-Zn** at room temperature in ethanol after 2 days (Figure 8). Conversely, the isomerization of **5-Zn** to **6-Zn** in acetone at room temperature takes place in ca. 3 h (Figure 9). Note that **5-Zn** and **6-Zn** are the only crystalline products that have been identified in the solid state upon solvent evaporation. The ^1^H NMR investigation of isomerization processes in *d*^6^-ethanol and *d*^6^-acetone (Figures S7 and S8) at room temperature did not help to rationalize the mechanism. Both spectra do not correspond to the solid-state structures of **5-Zn** and **6-Zn** isomers and are clearly different from those recorded in noncoordinating solvents (Figure 5). Upon dissolving the **6-Zn** isomer in *d*^6^-ethanol, the NMR spectra (Figure S7) display a set of three tbaoac proton signals that transform to another close set over time. In the spectra of the **5-Zn** isomer dissolved in *d*^6^-acetone (Figure S8), only one set of broad tbaoac proton signals is visible, shifting upfield as the transformation proceeds. However, several points can be made from the analysis of NMR data. First, the spectra are changing over time in both solvents. Second, the solvent molecules are obviously participating in the process. Finally, the isomerization processes in ethanol and acetone are going through two different pathways that are not reversed by one another. No matter what species exist in the solution, the evaporation of solvents results only in **5-Zn** or **6-Zn** compounds or a mixture thereof. No other phases, including solvates, have been crystallized out. It should be stressed that the precipitation of **5-Zn** in ethanol is likely a driving force for the isomerization of **6-Zn** in this solvent, while both isomers have good solubility in acetone. These observations strongly support the idea that two transformations take place along the different pathways, though those do not help to explain the solution structures as well as the role of solvent molecules in isomerization.

Low-temperature ^1^H NMR study of **6-Zn** and **5-Zn** in acetone confirmed the peak split at −60 °C (Figure S9) similar to that in chloroform. However, there is no transformation taking place at this temperature, as was confirmed by continuous recording of the spectra over time. According to variable temperature measurements, the transformation starts at around −40 °C. No such observations are available in ethanol due to the extremely low solubility of isomers.

Considering that the structural change from **6-Zn** to **5-Zn** involves, at least, breaking four M–O bonds and making two new bridging interactions (vide supra), the isomerization process is already complex enough. Since the transformation between two isomers takes place in solutions of coordinating solvents only, the solvent molecules do participate in the process. The donor solvents can simply attach to 5-coordinated Zn center/centers and/or can break the bridging Li–O and Zn–O bonds while making metal centers coordinatively unsaturated upon evaporation and departure from the structures. The existence of numerous possible intermediates makes it hard to envisage the isomerization processes and come up with suitable transformation models.

## Conclusions

We describe the synthesis and characterization of two heterobimetallic Li–Zn coordination isomers [Li_2_Zn_2_(tbaoac)_6_] that have been obtained by the same stoichiometric reaction in different organic solvents. These isomers share similar properties in the solid state but display sharp differences in solutions. Unlike many common cases of coordination isomers, this unique pair of molecules is not a product of ligand isomerism or a simple variation of coordinating atoms. Although the difference between isomeric structures of **6-Zn** and **5-Zn** can be simplistically represented by changing the coordination of two tbaoac ligands from the μ_3_- to μ_2_-type, in reality, the transformation requires a very complex rearrangement that involves breaking two of each Zn–O and Li–O bonds, while making a pair of new Li–O contacts. While there is no transformation between isomers in the solid state and solutions of noncoordinating solvents, it has been found that **5-Zn** can be quantitatively isomerized at room temperature to **6-Zn** in acetone, while the latter rearranges back in ethanol. Apparently, the coordinating solvent molecules play a major role in the isomerization process by either coordinating to 5-coordinated Zn sites or breaking the M–O bridging bonds in 6-coordinated Zn. However, the overall transformation process includes a number of possible intermediates and is too complex to envisage.

## Supporting Information Available

Experimental procedures, powder X-ray diffraction patterns, X-ray crystallographic procedures, IR, DART-MS, and ^1^H/^7^Li NMR spectra (PDF)
