# Supplementary material for: Trickier than It Looks: Isomerization between Five- and Six-Coordinated Zinc in Heterometallic Li2Zn2 Molecule
Source: Inorg Chem. 2024 Jun 21;63(27):12426–32. doi: 10.1021/acs.inorgchem.4c00634 (PMC11234357; doi:10.1021/acs.inorgchem.4c00634)
Supplement: Supplementary file 1 — ic4c00634_si_001.pdf [file ic4c00634_si_001.pdf]

Electronic Supplementary Information

Trickier than It Looks: Isomerization between Five- and Six-Coordinated Zinc in Heterometallic Li_2Zn_2 Molecule

Yuxuan Zhang,[a] Haixiang Han,[b] Zheng Wei,[a] Evgeny V. Dikarev[a]*

[a] Department of Chemistry, University at Albany, SUNY, Albany, NY 12222, United States
[b] School of Material Science and Engineering, Tongji University, Shanghai, 201804, China

*Author to whom correspondence should be addressed. E-mail : edikarev@albany.edu. Phone : (518)442-4401. Fax: (518)442-3462.

Table of Contents

1. General Procedures 3

2. Synthesis of Heterometallic Isomers 4

3. X-Ray Powder Diffraction Patterns of **5-Zn** and **6-Zn** Isomers 5

4. Single Crystal Growth 7

5. X-Ray Crystallographic Procedures 8

6. Solid State Structures of **5-Zn** and **6-Zn** Isomers 10

7. Chirality of Zn Centers in **5-Zn** and **6-Zn** Molecules 14

8. Direct Analysis in Real Time (DART) Mass Spectra of **5-Zn** and **6-Zn** Isomers 15

9. ^{1}H and ^{7}Li NMR Spectra of **5-Zn** and **6-Zn** Isomers in Different Solvents 16

10. ATR-IR Spectra of **5-Zn** and **6-Zn** Isomers 18

11. References 19

1. General Procedures

Tert-butyl acetoacetate (Htbaoac), $CDCl_3$, acetone-d^6, ethanol-d^6, DMSO-d^6 and D_2O were purchased from Sigma-Aldrich and used as received. Anhydrous zinc chloride and lithium methoxide were purchased from Sigma-Aldrich and used as received after checking their powder X-ray diffraction patterns. The NMR spectra were recorded on a Bruker Ascend-500 spectrometer (500 MHz for ^1H and 155.5 MHz for ^7Li). Chemical shifts (δ) are given in ppm relative to the residual solvent peaks for ^1H and to the ^7Li peak of external standard (0.1 M solution of LiCl in D_2O). The ICP-OES analyses were carried out on ICPE-9820 plasma atomic emission spectrometer, Shimadzu. The IR spectra were measured using a Shimadzu IRTracer-100 Fourier Transform Infrared Spectrophotometer. The DART-MS spectra were recorded on a JEOL AccuTof 4G LC-plus DART mass spectrometer over the mass range of *m/z* 50–2000 at one spectrum per second with a gas heater temperature of 300 °C. X-ray powder diffraction data were collected on a Rigaku multipurpose θ-θ X-ray SmartLab SE diffractometer (Cu Kα radiation, HyPix-400 two-dimensional advanced photon counting hybrid pixel array detector, step of 0.01° 2θ, 20 °C). Le Bail fit for powder diffraction patterns has been performed using TOPAS version 4 software package (Bruker AXS, 2006). Thermogravimetric (TGA) measurements were carried out under 25 mL/min N_2 protection flow at a heating rate at 0.1-1 °C/min using a TGA 5500 (TA Instruments-Waters LLC).

2. Synthesis of Heterometallic Isomers

5-Coordinated $Li_2Zn_2(tbaoac)_6$ (5-Zn)

A flask was charged with a mixture of $ZnCl_2$ (0.100 g, 0.734 mmol) and Li(tbaoac)[1] (0.361 g, 2.20 mmol) under dry argon atmosphere, and 15 mL of dry, oxygen-free ethanol was added. The colorless solution was stirred for 36 hours at room temperature to result in appearance of white precipitate. The precipitate was collected by filtration at room temperature and dried under vacuum at 100 ^oC sand bath overnight. The yield was *ca*. 0.360 g (90%). The purity of the crystalline product was confirmed by X-ray powder diffraction analysis (Figure S1 and Table S1). ICP-OES (2% HNO_3 water solution, 20 ^oC): Li, 1.30% (Calcd: 1.28%); Zn, 12.0% (Calcd: 12.0%). ^1H NMR (500 MHz, $CDCl_3$, 20 ^oC): δ = 1.399 (s, -C(CH_3)$_3$), 1.439 (s, -C(CH_3)$_3$), 1.868 (s, -CH_3), 1.933 (s, -CH_3), 4.694 (s, -C*H*), 4.765 (s, -C*H*). ^7Li NMR (155.5 MHz, $CDCl_3$, 20 ^oC): δ = 1.60 (s).

6-Coordinated $Li_2Zn_2(tbaoac)_6$ (6-Zn)

A flask was charged with a mixture of $ZnCl_2$ (0.100 g, 0.734 mmol) and Li(tbaoac)[1] (0.361 g, 2.20 mmol) under dry argon atmosphere, and 15 mL of oxygen-free acetone was added. The colorless solution was stirred for 12 hours at room temperature and then evaporated under vacuum. The white residue was further dried under vacuum at 100 ^oC sand bath overnight. Residue was then redissolved in degassed, dry hexanes, and the LiCl was filtered off. Solvent was then evaporated under vacuum at room temperature. The yield was *ca*. 0.370 g (93%). The purity of the crystalline product was confirmed by X-ray powder diffraction analysis (Figure S2 and Table S2). ICP-OES (2% HNO_3 water solution, 20 ^oC): Li, 1.30% (Calcd: 1.28%); Zn, 12.2% (Calcd: 12.0%). ^1H NMR (500 MHz, $CDCl_3$, 20 ^oC): δ = 1.368 (s, -C(CH_3)$_3$), 1.424 (s, -C(CH_3)$_3$), 1.715 (s, -CH_3), 1.800 (s, -CH_3), 4.713 (s, -C*H*), 4.896 (s, -C*H*). ^7Li NMR (155.5 MHz, $CDCl_3$, 20 ^oC): δ = 1.85 (s).

3. X-Ray Powder Diffraction Patterns of 5-Zn and 6-Zn Isomers

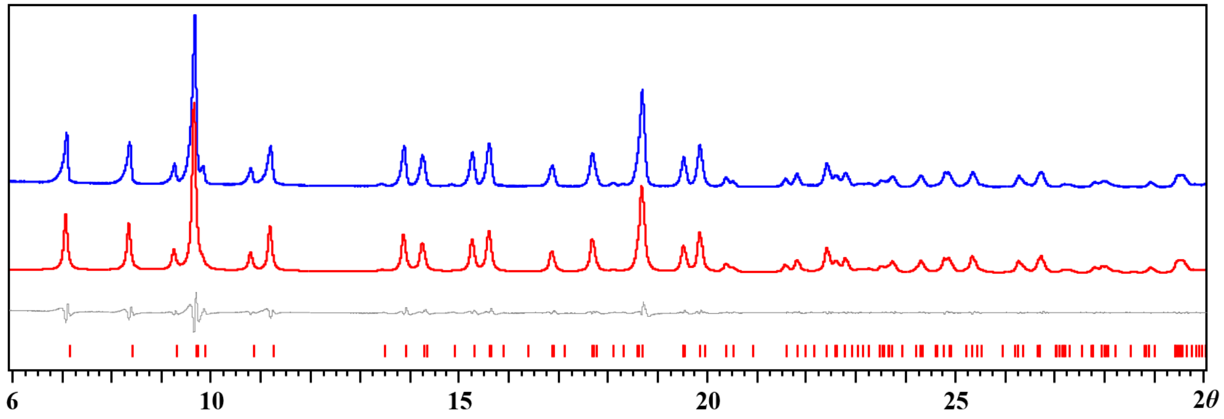

Figure S1. Powder diffraction pattern and Le Bail fit for the bulk powder of **5-Zn** isomer. Blue and red curves are experimental and calculated patterns, respectively, grey curve is the difference line. Theoretical peak positions are marked at the bottom as red bars.

Table S1. Unit cell parameters of **5-Zn** isomer obtained by the Le Bail Fit and from the single crystal data.

**5-Zn**		
	Le Bail Fit (20 °C)	Single Crystal Data (-173 °C)
Space Group	*P*-1	*P*-1
a (Å)	11.16614(19)	11.1559(11)
b (Å)	11.69369(17)	11.4249(12)
c (Å)	13.3097(2)	13.2169(13)
α (°)	78.8260(10)	78.4340(10)
β (°)	68.1330(11)	67.770(3)
γ (°)	63.9211(9)	63.875(2)
V (Å^3)	1447.71(4)	1398.9(2)

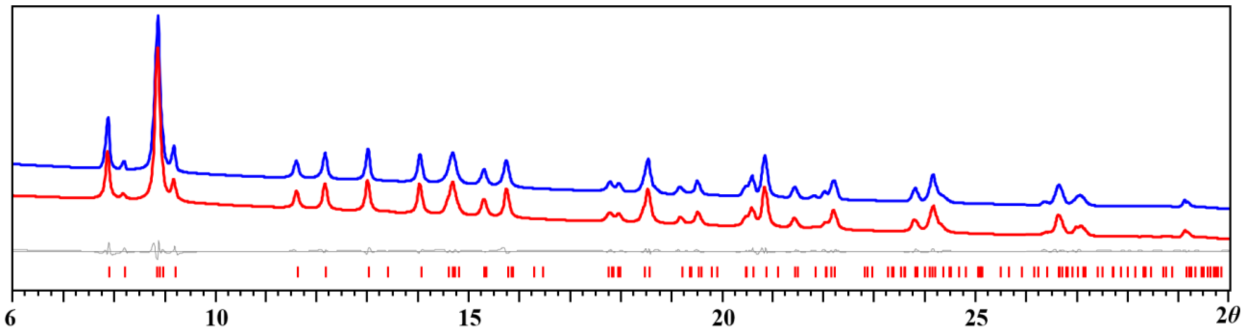

Figure S2. Powder diffraction pattern and Le Bail fit for the bulk powder of **6-Zn** isomer. Blue and red curves are experimental and calculated patterns, respectively, grey curve is the difference line. Theoretical peak positions are marked at the bottom as red bars.

Table S2. Unit cell parameters for **6-Zn** isomer obtained by the Le Bail Fit and from the single crystal data.

**6-Zn**		
	Le Bail Fit (20 °C)	Single Crystal Data (-173 °C)
Space Group	*P*-1	*P*-1
a (Å)	11.6749(4)	11.4282(4)
b (Å)	12.4761(4)	12.2357(5)
c (Å)	12.5792(5)	12.4450(6)
α (°)	104.044(2)	103.6680(10)
β (°)	105.986(2)	107.1850(10)
γ (°)	111.141(2)	110.850(2)
V (Å^3)	1518.64(11)	1435.53(11)

4. Single Crystal Growth

Single crystals of **5-Zn** and **6-Zn** isomers suitable for X-ray structural measurements were obtained by slow evaporation method at room temperature in the open air. Colorless, block-shaped crystals of **5-Zn** isomer were grown from its saturated dichloromethane solution in the glass tube for two weeks. Colorless, block-shaped crystals of **6-Zn** isomer were grown from its saturated acetone solution in the glass tube for about two weeks.

5. X-Ray Crystallographic Procedures

Single crystals of **5-Zn** and **6-Zn** isomers were mounted on glass fibers and cooled to 100(2) K using an Oxford Instruments Cryojet cryostat. The Bruker D8 diffractometer, integrated with a Pilatus 3×2M detector was modified for synchrotron use at the Chem-MatCARS 15-ID-B beamline at the Advanced Photon Source (Argonne National Laboratory). Diffraction data were collected at 30 keV with 0.5 s frames using ϕ scans, while manually attenuating the beam to minimize overages of individual pixels. Reduction and integration of collected data were performed with the Bruker APEX3 software package SAINT (version 8.37A).[2] The data were scaled and corrected for absorption effects using the multi-scan procedure as implemented in SADABS.[3] The structures were solved by SHELXT[4] and refined by a full-matrix least-squares procedures using the SHELXTL (version 2019/2)[5] software package through the OLEX2 graphical interface.[6] All non-hydrogen atoms were refined anisotropically. All hydrogen atoms were included in calculated positions and refined as riders, with $U_{iso}(H) = 1.2 \cdot U_{eq}(C)$ and $U_{iso}(H) = 1.5 \cdot U_{eq}(C)$ for methyl groups. All disorders found in these structures were modeled with two orientations. The geometries of the disordered parts were restrained to be similar. Anisotropic displacement parameters of the disordered fragments in the direction of the bonds were restrained to be equal with a standard uncertainty of 0.004 Å^2. Those were also restrained to have the same U_{ij} components, with a standard uncertainty of 0.01 Å^2. Crystallographic data and details of the data collection and structure refinement are listed in Table S3.

Table S3. Crystal Data and Structure Refinement Parameters for the **5-Zn** and **6-Zn** Isomers.

**Compound**	**5-Zn**	**6-Zn**
Empirical formula	$C_{48}H_{78}Li_2O_{18}Zn_2$	$C_{48}H_{78}Li_2O_{18}Zn_2$
CCDC number	2330865	2330866
Formula weight	1087.76	1087.76
Temperature (K)	100	100
Wavelength (Å)	0.41328	0.41328
Crystal system	Triclinic	Triclinic
Space group	*P*-1	*P*-1
a (Å)	11.1559(11)	11.4282(4)
b (Å)	11.4249(12)	12.2357(5)
c (Å)	13.2169(13)	12.4450(6)
α (°)	78.4340(10)	103.6680(10)
β (°)	67.770(3)	107.1850(10)
γ (°)	63.875(2)	110.850(2)
V (Å^3)	1398.9(2)	1435.53(11)
Z	1	1
ρ_{calcd} (g·cm^{-3})	1.291	1.258
μ (mm^{-1})	0.221	0.216
F(000)	576	576
Crystal size (mm)	0.09 × 0.06 × 0.04	0.15 × 0.12 × 0.11
θ range for data collection (°)	2.30–19.88	2.68–16.81
Reflections collected	75590	28598
Independent reflections	12674	8921
Transmission factors (min/max)	0.5380/0.6237	0.6038/0.7107
Data/restraints/params.	12674/57/359	8921/0/328
$R1$,[a] $wR2$[b] ($I > 2\sigma(I)$)	0.0310/0.0828	0.0397/0.0975
$R1$,[a] $wR2$[b] (all data)	0.0383/0.0866	0.0547/0.1021
Quality-of-fit[c]	1.030	1.030

[a]$R1 = \Sigma||F_o|-|F_c||/\Sigma|F_o|$. [b]$wR2 = [\Sigma[w(F_o^2-F_c^2)^2]/\Sigma[w(F_o^2)^2]]$.

[c]Quality-of-fit = $[\Sigma[w(F_o^2-F_c^2)^2]/(N_{obs}-N_{params})]^{½}$, based on all data.

6. Solid State Structures of 5-Zn and 6-Zn Isomers

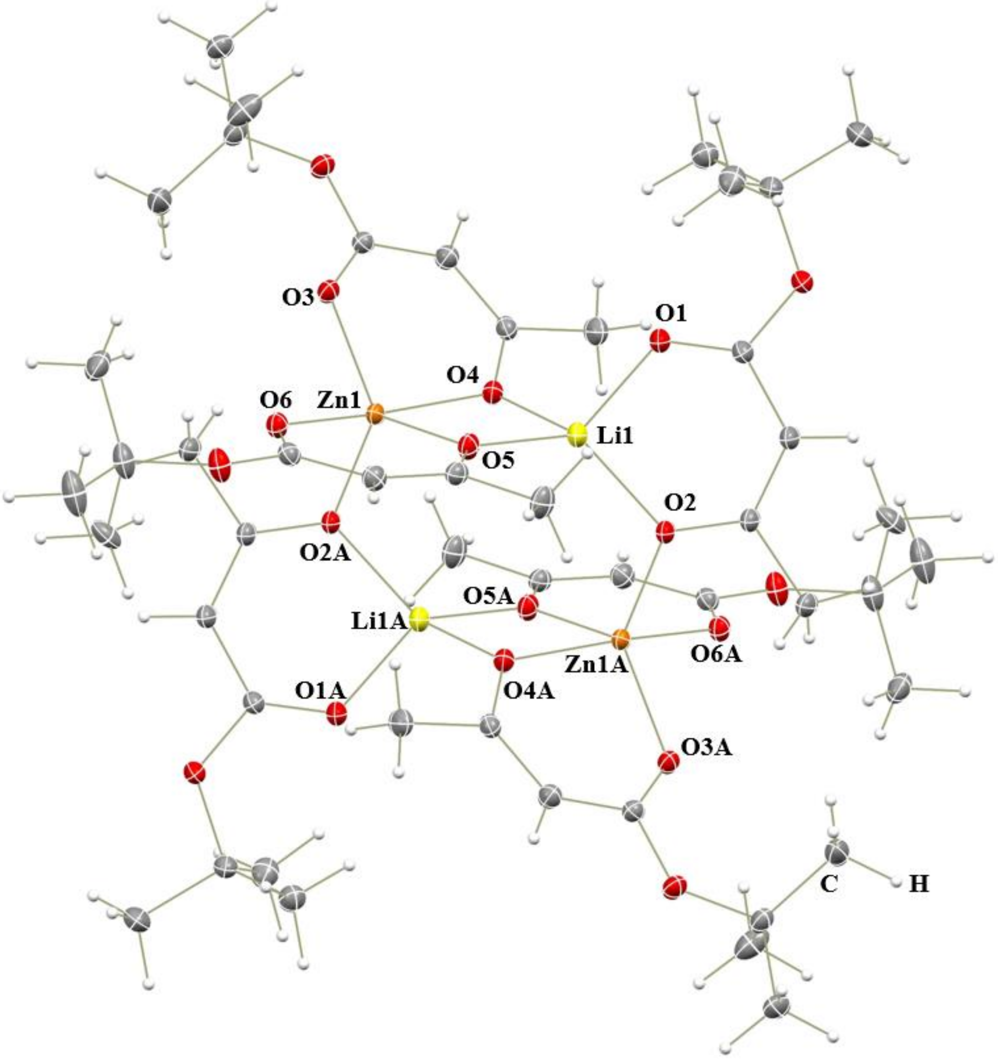

Figure S3. Solid-state structure of **5-Zn** isomer drawn with thermal ellipsoids at the 30% probability level. Hydrogen atoms are represented by spheres of arbitrary radius.

Table S4. Bond Distances (Å) and Angles (deg.) in the Structure of **5-Zn** Isomer.

**Bond distances**					
Zn(1)–O(2A)	1.9956(7)	Li(1)–O(1)	1.8975(19)		
Zn(1)–O(3)	2.0177(6)	Li(1)–O(2)	1.9293(18)		
Zn(1)–O(4)	2.0472(7)	Li(1)–O(4)	1.9641(18)		
Zn(1)–O(5)	1.9988(7)	Li(1)–O(5)	1.9216(19)		
Zn(1)–O(6)	2.0728(7)				
**Angles**					
O(2A)–Zn(1)–O(3)	116.65(3)	O(3)–Zn(1)–O(6)	92.08(3)	O(1)–Li(1)–O(2)	96.78(8)
O(2A)–Zn(1)–O(4)	97.54(3)	O(4)–Zn(1)–O(6)	165.53(3)	O(1)–Li(1)–O(4)	110.43(9)
O(2A)–Zn(1)–O(5)	112.82(3)	O(5)–Zn(1)–O(3)	130.36(3)	O(1)–Li(1)–O(5)	117.83(10)
O(2A)–Zn(1)–O(6)	94.21(3)	O(5)–Zn(1)–O(4)	79.49(3)	O(2)–Li(1)–O(4)	129.33(10)
O(3)–Zn(1)–O(4)	90.18(3)	O(5)–Zn(1)–O(6)	88.19(3)	O(5)–Li(1)–O(2)	120.68(10)
				O(5)–Li(1)–O(4)	83.48(7)

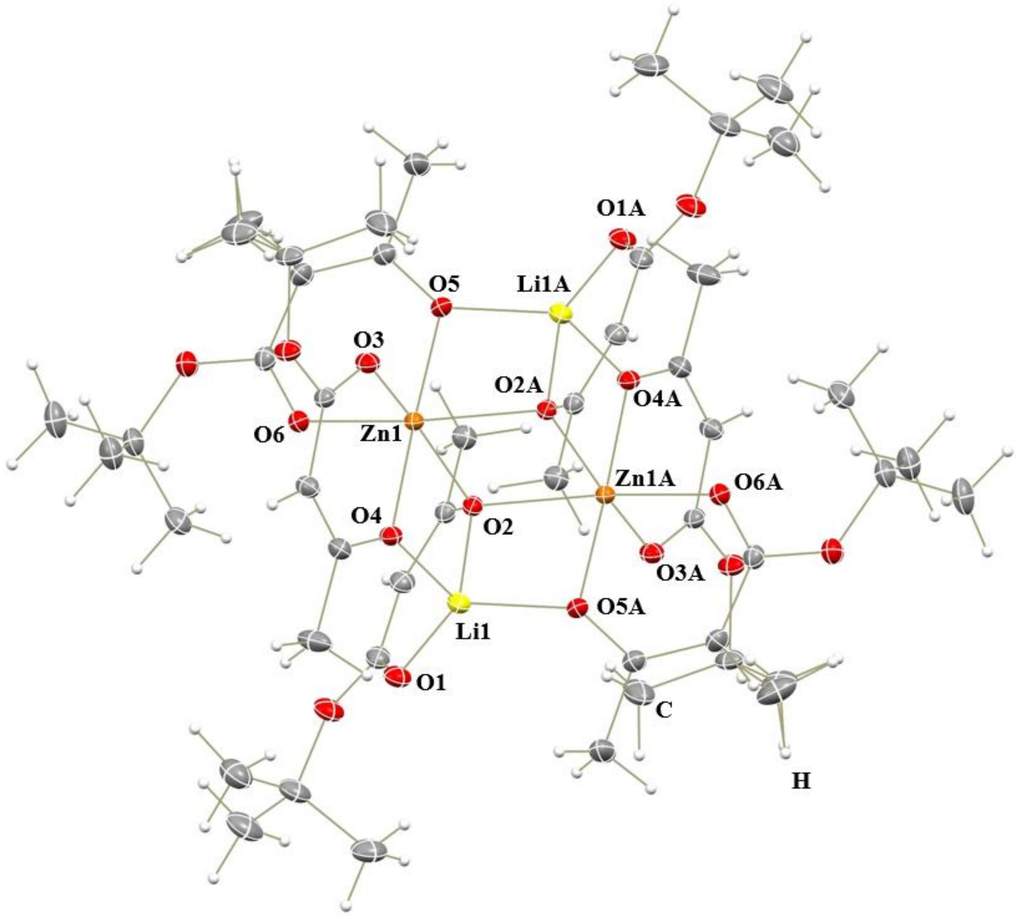

Figure S4. Solid state structure of **6-Zn** isomer drawn with thermal ellipsoids at the 30% probability level. Hydrogen atoms are represented by spheres of arbitrary radius.

Table S5. Bond Distances (Å) and Angles (deg.) in the Structure of **6-Zn** Isomer.

**Bond distances**					
Zn(1)–O(2)	2.1487(12)	Li(1)–O(1)	1.842(3)		
Zn(1)–O(2A)	2.1805(12)	Li(1)–O(2)	1.938(3)		
Zn(1)–O(3)	2.0466(13)	Li(1)–O(4)	1.883(3)		
Zn(1)–O(4)	2.0214(12)	Li(1)–O(5A)	1.872(3)		
Zn(1)–O(5)	2.0254(12)				
Zn(1)–O(6)	2.0400(12)				
**Angles**					
O(2)–Zn(1)–O(2A)	80.72(5)	O(4)–Zn(1)–O(6)	93.29(5)	O(1)–Li(1)–O(2)	100.04(16)
O(3)–Zn(1)–O(2)	167.05(5)	O(5)–Zn(1)–O(2A)	97.84(5)	O(1)–Li(1)–O(4)	114.72(17)
O(3)–Zn(1)–O(2A)	94.19(5)	O(5)–Zn(1)–O(2)	81.37(5)	O(1)–Li(1)–O(5A)	124.85(18)
O(4)–Zn(1)–O(2)	96.90(5)	O(5)–Zn(1)–O(3)	93.12(5)	O(4)–Li(1)–O(2)	91.23(14)
O(4)–Zn(1)–O(2A)	80.99(5)	O(5)–Zn(1)–O(6)	88.29(5)	O(5A)–Li(1)–O(2)	91.21(14)
O(4)–Zn(1)–O(3)	87.86(5)	O(6)–Zn(1)–O(2A)	166.29(5)	O(5A)–Li(1)–O(4)	118.83(17)
O(4)–Zn(1)–O(5)	178.06(5)	O(6)–Zn(1)–O(2)	91.88(5)		
		O(6)–Zn(1)–O(3)	95.34(5)		

7. Chirality of Zn Centers in 5-Zn and 6-Zn Molecules

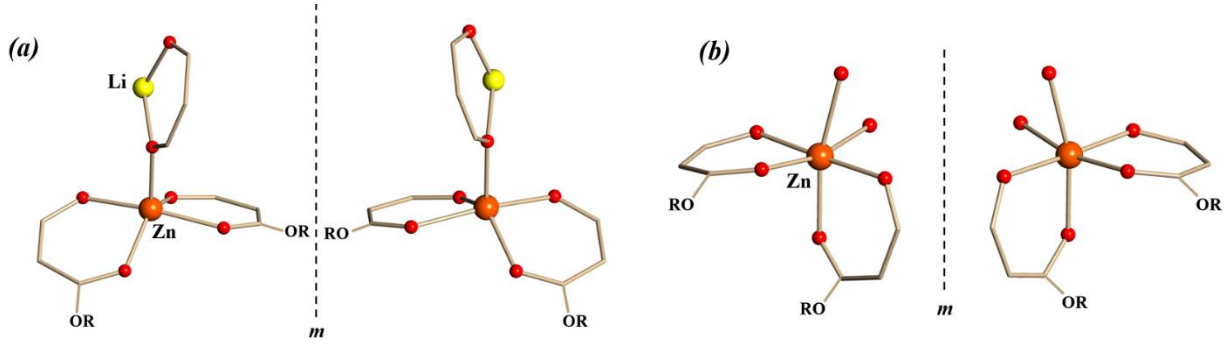

Figure S5. Chirality of Zn centers in a) **5-Zn** isomer and b) **6-Zn** isomer.

8. Direct Analysis in Real Time (DART) Mass Spectra of 5-Zn and 6-Zn Isomers

Table S6. Assignment of Ions Detected in Positive-Ion DART Mass Spectrum of **6-Zn** Isomer.

Species	*Observed*	*Calculated*	*Δ*	*% Base*
**$LiZn_3(tbaoac)_6^+$**	1146.251	1146.250	0.0001	26.5
**$Li_3Zn_2(tbaoac)_6^+$**	1094.743	1094.735	0.0003	13.3
**$Zn_3(tbaoac)_5^+$**	982.120	982.123	0.0002	60.6
**$Li_2Zn_2(tbaoac)_5^+$**	930.612	930.608	0.0005	15.2
**$LiZn_2(tbaoac)_4^+$**	766.552	766.481	0.0010	5.5
**$Zn_2(tbaoac)_3^+$**	602.350	602.353	0.0006	17.0
**$HLiZn(tbaoac)_3^+$**	544.909	544.906	0.0006	7.9
**$LiZn(tbaoac)_2^+$**	386.711	386.710	0.0001	100
**$Li_3(tbaoac)_2^+$**	335.150	335.195	0.0025	20.2

Table S7. Assignment of Ions Detected in Positive-Ion DART Mass Spectrum of **5-Zn** Isomer.

Species	*Observed*	*Calculated*	*Δ*	*% Base*
**$LiZn_3(tbaoac)_6^+$**	1146.245	1146.250	0.0004	1.6
**$Li_3Zn_2(tbaoac)_6^+$**	1094.720	1094.735	0.0010	8.1
**$Li_5Zn(tbaoac)_6^+$**	1043.292	1043.220	0.0002	1.7
**$Zn_3(tbaoac)_5^+$**	982.120	982.123	0.0001	4.9
**$Li_2Zn_2(tbaoac)_5^+$**	930.605	930.608	0.0025	7.0
**$Li_4Zn(tbaoac)_5^+$**	879.085	879.092	0.0033	7.6
**$Zn_3(tbaoac)_4^{2+}$**	824.929	824.936	0.0005	4.4
**$LiZn_2(tbaoac)_4^+$**	766.354	766.481	0.0032	4.3
**$Li_3Zn(tbaoac)_4^+$**	714.965	714.965	0.0015	7.1
**$HLi_2Zn(tbaoac)_4^+$**	708.905	709.033	0.0017	3.6
**$Zn_2(tbaoac)_3^+$**	602.350	602.353	0.0001	10.2
**$HLiZn(tbaoac)_3^+$**	544.805	544.906	0.0044	7.7
**$LiZn(tbaoac)_2^+$**	386.597	386.710	0.0026	38.5
**$Li_3(tbaoac)_2^+$**	335.104	335.195	0.0009	100

9. ^{1}H and ^{7}Li NMR Spectra of 5-Zn and 6-Zn Isomers in Different Solvents

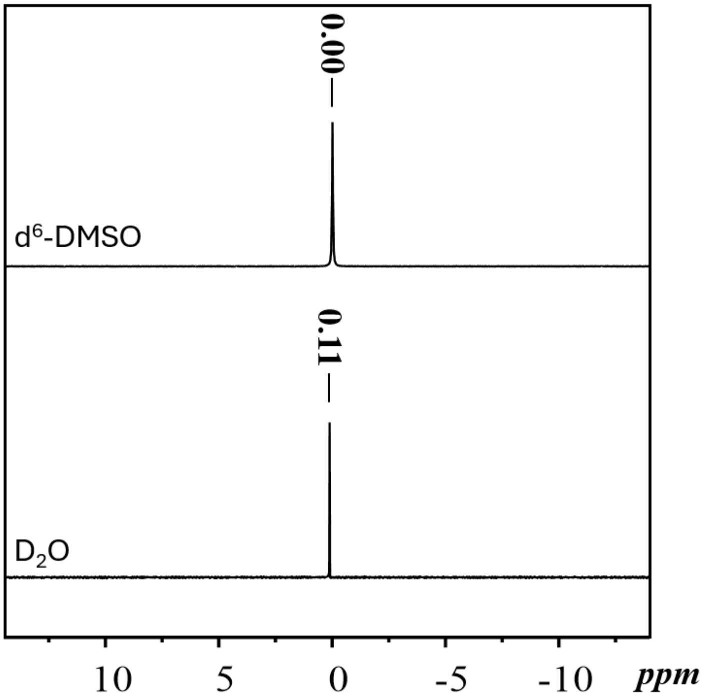

Figure S6. ^{7}Li NMR spectra of **6-Zn** in d^6-DMSO and D_2O at room temperature.

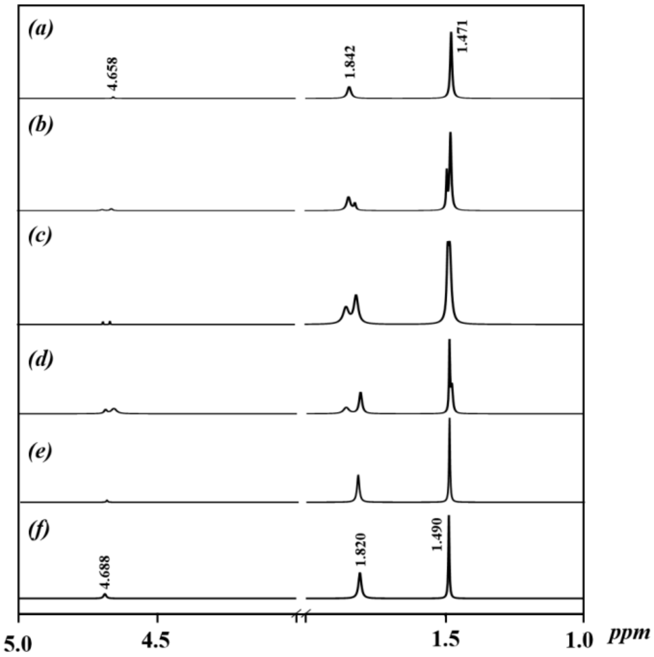

Figure S7. ^{1}H NMR spectra in dry *d*^{6}-ethanol recorded at room temperature: *a*) **6-Zn** immediately upon dissolution; *b*) after 1 hour; *c*) after 6 hours; *d*) after 12 hours; *e*) after 2 days; *f*) **5-Zn** immediately upon dissolution.

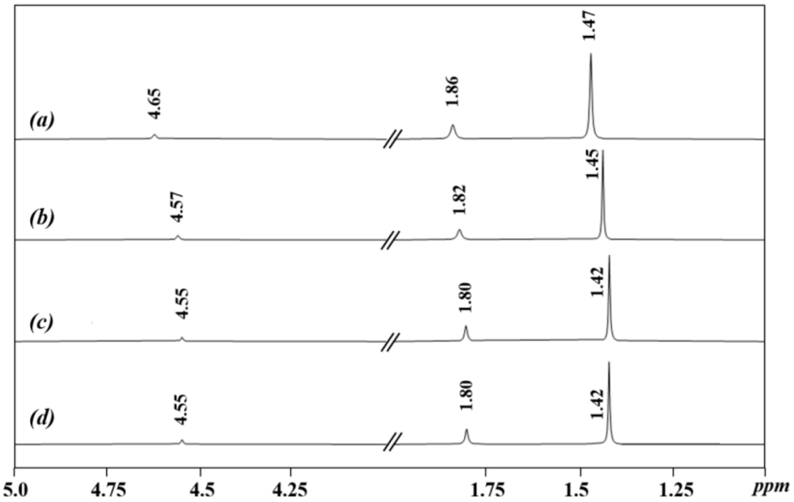

Figure S8. ^{1}H NMR spectra in dry d^6-acetone recorded at room temperature: *a*) **5-Zn** immediately upon dissolution; *b*) after 30 minutes; *c*) after 3 hours; *d*) **6-Zn** immediately upon dissolution.

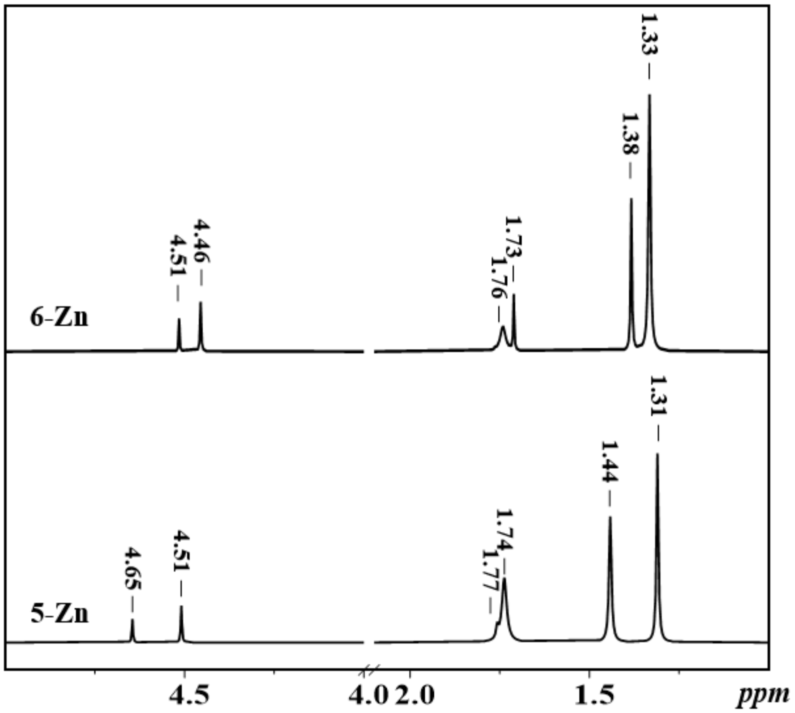

Figure S9. ^{1}H NMR spectra of **6-Zn** (up) and **5-Zn** (down) in d^6-acetone at -60 ^{o}C.

10. ATR-IR Spectra of 5-Zn and 6-Zn Isomers

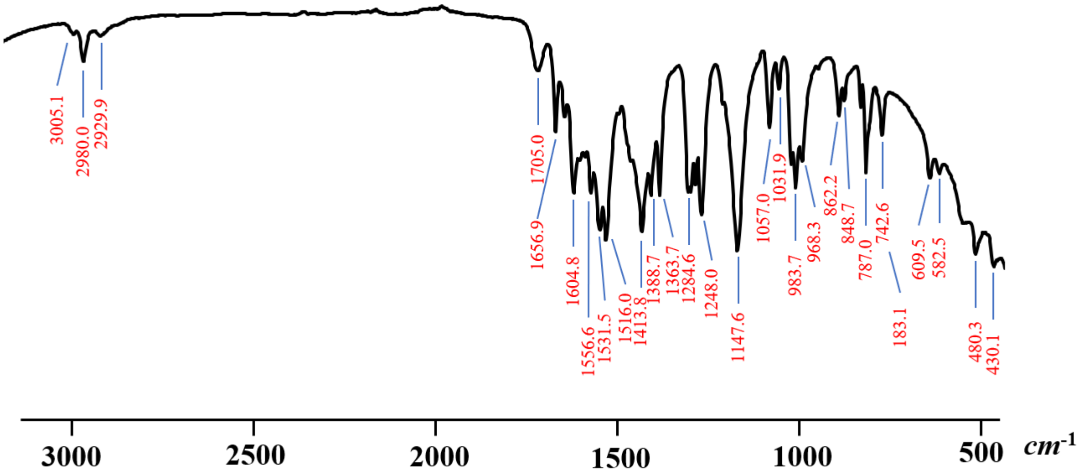

Figure S10. The attenuated total reflection (ATR) spectrum of **5-Zn** isomer.

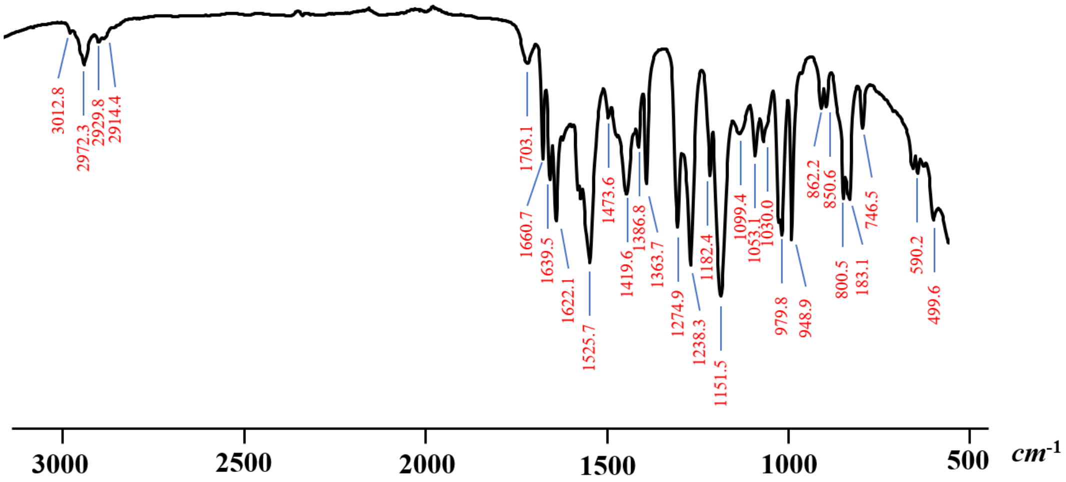

Figure S11. The attenuated total reflection (ATR) spectrum of **6-Zn** isomer.

11. References

1. Wei, Z.; Han, H.; Filatov, A.S.; Dikarev, E.V. Changing the Bridging Connectivity Pattern within a Heterometallic Assembly: Design of Single-Source Precursors with Discrete Molecular Structures. *Chem. Sci*. **2014**, *5*, 813–818.
2. *SAINT*, version 2017.3-0; Part of Bruker APEX3 Software Package; Bruker AXS: Billerica, MA, USA, **2017**.
3. *SADABS*, version 2017.3-0; Part of Bruker APEX3 Software Package; Bruker AXS: Billerica, MA, USA, **2017**.
4. Sheldrick, G.M. SHELXT—Integrated Space-Group and Crystal-Structure Determination. *Acta Crystallogr. A Found. Adv*. **2015**, *71*, 3–8.
5. Sheldrick, G. M. Crystal Structure Refinement with SHELXL. *Acta Crystallogr. C Struct. Chem.* **2015**, *71*, 3–8.
6. Dolomanov, O. V.; Bourhis, L. J.; Gildea, R. J.; Howard, J. A. K.; Puschmann, H. OLEX2: A Complete Structure Solution, Refinement and Analysis Program. *J. Appl. Crystallogr*. **2009**, *42*, 339–341.
